# Deontological Feeling: The Tranquil, the Familiar and the Body

**DOI:** 10.3389/fpsyg.2021.662675

**Published:** 2021-06-29

**Authors:** Henning Nörenberg

**Affiliations:** Department of Philosophy, University of Rostock, Rostock, Germany

**Keywords:** existential feeling, deontic power, affordance, situated normativity, ethical foundations, qualitative research

## Abstract

This paper contributes to filling a lacuna in recent research on common normative backgrounds. On the one hand, discussions of common normative backgrounds tend to underexpose the role the feeling body plays in relation to the agent’s recognition of deontic powers (obligations, compelling reasons or rights). On the other hand, discussions of bodily background orientations and their role in the agent’s sensitivity to practical significance tend to underexpose the recognition of deontic power. In this paper, I argue that bodily background orientations can contribute to an agent’s sensitivity to deontic power. Developing further on Ratcliffe’s conceptualization of existential feelings, I propose that a person’s bodily background orientation implies responsiveness to an ethically significant kind of affordance. In order to flesh out this theoretical claim, I draw on empirical material concerning a specific existential orientation labelled as “quietism.” Reconstructing its central patterns, I explicate the bodily dimension involved in the quietist orientation as well as the way in which it shapes the responsiveness to felt demands in terms of preserving tranquillity and protecting the familiar. Finally, I discuss the broader theoretical implications of my claim and suggest to categorize ethically relevant bodily background orientations such as the one implicated in the quietist orientation as deontological feelings.

## Introduction

A vast range of phenomena, such as cultural conflict, echo chambers and increasing political divides in Western societies, points to the fact that practical reasoning can only function where there is a common normative background. Only against such a background can something emerge as a compelling reason, as fair, as demanding my or our respect, and as my or our obligation or right. For instance, you and me cannot have a rational debate about how to mitigate global warming, unless both of us recognise global warming as a fact and also as a problem we can and ought to engage with.

In part, the background in question can be elucidated by pointing out particular norms that, at least in principle, could be codified, e.g., formal conditions regarding noncontradiction, ideals, values, projects and many more (Habermas, 1984). Many accounts also acknowledge that such common normative backgrounds are, in part, structured by elements pertaining to the bodily orientation of an agent. Searle, for instance, has argued that obligations and rights – “deontic powers,” as he calls them – have the logical structure of declarative speech acts and motivate the agent by providing them with desire-independent reasons for action (see Searle, 2010, 81, 127 f.). According to Searle, the recognition of such deontic powers depends on what he calls the background, which is, among other things, constituted by affective styles, motor capacities, modes of sensibility and sensorimotor experience. Yet these and various other accounts pointing in a similar direction (Bourdieu, 1990; Lakoff, 1996; McDowell, 1998; Haidt et al., 2009) predominantly focus on elements like tacit assumptions, social practices, concerns, symbols, narratives, etc. They do not fully explore the bodily-affective dimension involved in the agent’s recognition of something as their right or obligation.

Alternatively, there are accounts that point out a constitutive link between the bodily condition of the agent and the practical significance with which the agent’s world is imbued. The pivotal point of these accounts is the notion of an ecstatic and feeling body as spelled out in various pragmatist, phenomenological and enactivist accounts (Leder, 1990; Merleau-Ponty, 2000; Gallagher, 2005; Ratcliffe, 2008; Slaby, 2008; Colombetti, 2014). The ecstatic, feeling body is attuned to the surrounding world in terms of a kinaesthetic background sense of instrumental possibilities (“I/you/them can/cannot φ”) or aesthetic tensions (“This does not feel right yet”) that make up a complex structure of affordances (Gibson, 1979, 127 ff.; Dreyfus and Kelly, 2007, 52 ff.; Rietveld, 2008, 980 ff.; Withagen et al., 2012, 253 ff.). Although there are some initial attempts to connect bodily orientations with morally relevant normative evaluations (Ratcliffe, 2010; Slaby, 2012; Bortolan, 2017), the discussion of bodily background orientation has, so far, mainly focused on instrumental and aesthetic possibilities, whereas the sense of practical normativity in the form “I/you/them ought (not) to φ” is widely underexplored.

Thus, apparently, there is a *double lacuna*: While the discussion of bodily background orientations and their role in the agent’s sensitivity to practical significance tends to underexpose “oughtness,” the discussion of common normative backgrounds tends to underexpose the role of the feeling body in relation to the agent’s recognition of obligations, compelling reasons or rights. But is there not a bodily orientation towards the world involved in one’s predisposition, for instance, to refrain from taking the initiative in particular situations, not only because one has the sense that one cannot do it but rather because one feels one ought not to do it? Or, more broadly, is there not a connection between one’s bodily background orientation and the kind of norms one accepts as binding? I suggest there is.

In order to show how bodily background orientations contribute to a person’s sensitivity to obligations, I draw on a qualitative empirical investigation that was part of the research project “Sense of Life and Politics in Mecklenburg-Western-Pomerania” to which I contributed. The original focus of this investigation was on a connection between a region-typical “sense of life,” i.e., a specific way of finding oneself in the world, and regionally predominant political attitudes. The focus of the present paper is on the bodily dimension involved in this way of finding oneself in the world and the sensitivity to particular forms of deontic power it implies, *the deontological aspect of a bodily background orientation*, if you will.

More precisely, I suggest conceiving of the aspect in question in terms of an ethically predisposing bodily attunement to a particular quality pervading the lived environment of an individual.^1^ The attunement in the focus of this paper is characterised by the agent’s responsiveness to the demand for preserving the tranquillity they perceive in their environment, by a preference to remain in the familiar social sphere, and by depreciating any adaptation to conditions beyond that sphere as idle “pretence.” As a label for this attunement, I suggest “quietism” as this term at least in part captures some relevant traits of the attunement.^2^ The argument unfolds as follows. After having outlined the theoretical context of this investigation, I explain the methods used for gathering and interpreting the empirical data. Then, interpreting the empirical material, I present a reconstruction of the quietist attunement, its bodily dimension, as well as its role in shaping the relevant participants’ sensitivity to deontic power. Finally, I discuss the broader theoretical implications of the analysis by outlining the way in which it may be warranted to categorise the quietist orientation as a *deontological feeling*.

## Affordance-Borne Normativity, Deontic Power, and the Body

Central insights of both the research on bodily background orientations and normative backgrounds can be combined in an ethically significant notion of *affordance-borne normativity* (Rietveld, 2008; Fuchs, 2019; Nörenberg, 2020; see also Mandelbaum, 1955; Schmitz, 1973). The aim of this section is to outline that notion in connection with several other concepts that will be relevant to understanding the quietist attunement.

Before that, a clarificatory remark is appropriate. This investigation is centrally concerned with the way in which an agent understands a particular norm. For instance, I may abstractly understand and judge that I ought to give you back those five dollars I promised you, yet fail to act accordingly. Rather than concentrating on inconsistencies and gaps between my actions and the content of my ought-judgment, the current approach suggests paying more attention to the overall experiential event or the particular way in which I find myself in the relevant situation, including the content of the judgement, the way it is formed, the action and affects (see Williams and Gantt, 2012, 424). On this perspective, it is the way that I understand my commitment of giving the money back to you that is the problem, since it allows – perhaps in a subjectively consistent manner – for various ways for me to neglect my promise. That is, while I formally recognise the obligation that comes with the promise I made, I may just not *feel* the respective demand and, in this sense, fail to genuinely understand and recognise the norm as ethically binding.^3^ Likewise, I may feel ethically bound to a norm, the objective validity of which is, in the eyes of a third person, debatable or even denied. Thus, in the context of the present investigation, we should distinguish the ethical bindingness of a norm from its objective validity. What is intended by ethical bindingness or “oughtness” is more closely related to the perspective of the agent: What is it that the agent understands and recognises as their or another person’s obligation or right, etc., and how is it recognised by them?^4^

The kind of normativity that is relevant to this discussion is constituted by a particular type of soliciting *affordances*. In many cases, affordances are not merely there as practical possibilities neutrally “offered” by the environment (which is roughly the way in which Gibson thinks of them; see Gibson, 1979, 127, 139). Often, affordances are even experienced as “solicitations” in that they “invite” or “solicit” the relevant action so that the agent feels attracted or repelled by some affordances in their environment, e.g., kicking a ball lying in their path or avoiding a puddle (Dreyfus and Kelly, 2007, 52; Withagen et al., 2012, 257).

Before I further expose the *differentia specifica* of that affordance-borne normativity (ethical “oughtness”), some theoretical implications regarding solicitations in general need to be considered. The agent is aware of the complex structure of affordances in their relevant environment in terms of their *bodily background orientation* towards that environment, that is, in terms of their sense of there being certain kinaesthetic possibilities, nonconceptual, habitual bodily expectations, felt tensions, possibilities of bodily interaction, etc. (Ratcliffe, 2008, 44; 121 ff.; 155 f.). The background sense of the possibilities afforded by the environment shapes the way in which particular things “appear to us as inviting, valuable, fascinating, threatening, dull, repulsive, proper, improper, comforting, terrifying and so on” (Ratcliffe, 2009, 350). On the same grounds, even the world itself as a whole may appear as “familiar,” “homely,” “cosy,” “suffocating” or as something from which one feels detached (see Ratcliffe, 2008, 135 f.). These latter descriptions relate to a long-term background orientation of the relevant agent, which is independent from the particular situation and, therefore, called “existential orientation” or – because of its peculiar phenomenology – *existential feeling* (Ratcliffe, 2008, 55). Against the backdrop of specific existential feelings, particular intentional acts are supposed to receive or lose their intelligibility. For instance, without having at least a vague sense of there being very general potential for things to “turn out for the good,” any intentional state of the type “hope” would be ultimately unintelligible to one (Ratcliffe, 2013, 603).

This notion of bodily background orientations is related to, for instance, “temperaments” that predispose the subject to particular kinds of philosophical ideas or “fundamental attunements,” without which the proper meaning of particular concepts could not be grasped (James, 1907, 3 ff.; Heidegger, 1995, 5 ff., 59 ff.; 1996, 172 ff.; see Ratcliffe, 2008, 52 ff., 244 ff.). It is also related to Dewey’s notion of a single *pervasive quality* that is “felt rather than thought” and against the backdrop of which alone objects and their attributes are meaningful.^5^ That is, bodily background orientations as ways of finding oneself in a particular environment (or “situation” in Dewey’s terminology) entail a holistic sense of the character of that environment, a sense of how it appears (threatening, dull, homely, etc.). Although Dewey obviously does not refer to the term “affordance,” he intends to make a similar point when he argues that the experience of pervasive qualities is underpinned by practical possibilities and expectations (Dewey, 1931, 106). Both notions, bodily background orientation and pervasive quality, highlight different aspects of the same phenomenon. In the following, I refer to orientations or attunements in order to highlight the aspect of finding oneself in a particular environment. Whenever the aspect of there being a palpable character of the environment is to be highlighted, I refer to qualities.

Generally, affordances as solicitations imply a sense of normativity (Rietveld, 2008). For instance, a fruit in your neighbour’s garden may solicit reaching over the fence and, as such, imply a sense of appropriateness in terms of the optimal way of fulfilling the action. The sense of appropriate conduct in a given situation insofar as it is delineated in terms of solicitations – *situated normativity* as Rietveld (2008, 985 ff.) calls this – is holistic: How, for instance, the tacitly perceived requirement to maintain an appropriate distance from others in an elevator (Dreyfus, 2002, 417) is fulfilled in the optimal way, depends on the agent’s cultural background, on their sensitivity, as well as on the particular situation itself, e.g., on how many people there are in the elevator, but cannot be exactly specified in terms of a set of codifiable rules (Rietveld, 2008, 988; see also McDowell, 1998, 67). If you will, such affordance-borne situated normativity is an integral aspect of the pervasive quality of the situation.

However, in contrast to the examples just given, the particular kind of normativity in the focus of the present investigation does neither refer to instrumental nor to aesthetic norms. It is a distinct kind of practical normativity, that of ethical oughtness. The optimal way of reaching the fruit in your neighbour’s garden is usually but one aspect of affordance-borne situated normativity. For instance, the aspect that the fruit is behind your neighbour’s fence may also imply a vague sense of constraint, an impulse to leave it where it is. Among other things, this kind of normativity differs from the merely instrumental kind in terms of the sort of feelings it involves. While instrumental and aesthetic kinds of solicitations seem to involve feelings such as the “directed (dis)content” or satisfaction that underpins the situated normativity guiding the work of Wittgensteinian craftsmen (Rietveld, 2008, 980 ff.; see Wittgenstein, 1978), *oughtness* involves actual or at least affectively anticipated forms of ethically relevant feelings such as shame, resentment or indignation.^6^ The vague sense of constraint or shrinking back, for instance, that may hamper your otherwise optimal reach for the fruit behind the fence is the affective anticipation of – or felt preparedness for – the shame that is about to assail you if you realise what you are doing in the light of the look of another person.^7^ Similarly, your subtle sense of irritation regarding a person standing slightly too close to you in an otherwise empty elevator may phenomenologically differ from mere discontent and constitute a subtle form of resentment, which renders the disturbance of your personal space *illegitimate* rather than simply unpleasant. Whether the actual or anticipated feeling is shame-like or resentment-like depends on who actually violated – or is on the point of violating – the respective norm. Generally, fully actualised or affectively anticipated shame-like feelings are involved in recognising a norm imposed on oneself, while actualised or anticipated resentment-like feelings (including indignation and some forms of anger) are involved in one’s recognition that a norm imposed on others is ethically binding (though not or not yet met; see Landweer, 2011, 61).

Affordance-borne normativity in the sense outlined can link the discussions of bodily background orientations and normative backgrounds in a fruitful way. This normativity is, I suggest, involved in the agent’s recognition of an object’s capacity to give that agent desire-independent reasons for action (Searle, 2010, 123); for instance in the form of “rights, duties, obligations, commitments, authorizations, requirements, permissions and privileges” (Searle, 2007, 98). Searle calls this capacity *deontic power*. Deontic power means that there is something implicated in the way in which we relate to, for instance, the fence of our neighbour that prohibits us from simply climbing or reaching over it without the permission of our neighbour. In order to recognise the norm of leaving the property of our neighbour in peace not merely in terms of external sanctions or rules of prudence, but in terms of an obligation, I contend, the relevant demand must be felt accordingly. Insofar as deontic powers exist as long as they are recognised by the agent, the feeling of the demand as a condition for their appropriate recognition can help elucidate bodily background orientations as a relevant dimension of normative backgrounds, precisely because bodily background orientations consist in particular kinds of responsiveness to demands as solicitations.

To be sure, notions such as affordance, existential feeling and deontic power, originate in different conceptual frameworks, which, in turn, involve different theoretical commitments. Originally, the concept of affordance refers to affectively neutral opportunities for action and was distinguished from what gestalt psychologists called the “demand character” (see Koffka, 1935); it is only later that the soliciting power of (some) affordances re-emerges in the discussion (see Dreyfus and Kelly, 2007; Withagen et al., 2012; Ramstead et al., 2016; Rietveld and Brouwers, 2017; Weichold, 2018). Still, on few occasions, Ratcliffe suggests that even this differentiated notion of affordance would not exhaust what he calls the space of possibilities, the sense of which is supposed to set up existential feeling. Practically relevant possibilities do not only consist of attractive opportunities of action but also, for instance, of anticipation of what could happen to oneself or others, what is to be avoided, etc. (see Ratcliffe, 2008, 128). Nevertheless, many recent accounts of affordance structures seem to integrate practical possibilities in this broader sense, including that of situated normativity on which I am drawing in this investigation (e.g., Rietveld, 2008). Beyond that, Searle grounds his social ontology and his account of deontic power as status functions imposed on certain objects or persons in a particular kind of internalism according to which meaning is only in the brain (2010, 44). In contrast, one of the fundamental assumptions of the present investigation is that meaning is constituted in the individuals’ relation to their environment. Still, in the present context, Searle’s socio-ontological account is valuable as an attempt of explicating logical structures, although not as an attempt to explain how human cognition works in detail. So, despite these tensions, I think there is a fruitful way of combining central insights from these different frameworks in order to flesh out the bodily background sense of ethical normativity.

Previous accounts have already thematised the ethical dimension of existential bodily feelings of guilt, anger and shame in psychopathology (see Ratcliffe, 2010; Bortolan, 2017). Although these accounts suggest that they can also help elucidate more common forms of ethically predisposing bodily background orientations, such elucidation still is, by and large, an unresolved task (see also Ratcliffe, 2020, 258 f.). The present investigation into the quietist attunement is a contribution to that task.

## Background and Research Question

My consideration of quietism as a deontological background orientation refers to empirical material collected as a part of the research project “Sense of Life and Politics in Mecklenburg-Western-Pomerania.” The original intent of this part of the project was to investigate whether there is an existential orientation (“sense of life”) that is characteristic to the people in the north-eastern region of Germany and in which way the political views commonly held in the region may be connected with that existential orientation.^8^ The region in question is, as compared with other parts of Germany, characterised by a weak infrastructure, a low population density, high outflux rates, a high number of older people and an above-average rate of unemployment. Its landscape is predominantly made up of large fields, wooded areas and water. Politically, the region is organised as one of the 16 states within the Federal Republic and called “Mecklenburg-Western-Pomerania.”

As compared with the original intent of the project, the present investigation is not focused on reconstructing an existential orientation representative for a particular region but exemplarily investigates the way in which bodily background orientations imply sensitivity to deontic power. Thus, the material collected is analysed and interpreted with regard to reconstructing homological patterns in some of the participants’ existential orientations, explicating the bodily dimension implicated in those patterns and indicating the way in which that bodily dimension is involved in shaping the sensitivity to deontic power.

## Methodology

The empirical material was collected in 26 in-depth interviews in 2012 and 2013.^9^ The interviews were conducted by trained psychologists from the Rheingold Institute based in Cologne. The material then was analysed and interpreted in collaboration between psychologists (the interviewers themselves as well as other colleagues from their institute) and philosophers from the University of Rostock with a specialisation in phenomenology and social ontology.

The guiding methodological assumptions were these:

- The relevant elements of a regionally dominant “sense of life” are, to various extents, manifested in the individual existential orientations of the participants.
- Phenomena, such as existential orientations, are rather implicit and cannot be directly polled but have to be reconstructed in a collective, *multilevel hermeneutical process*.

The general qualitative approach aimed for exploring how a particular “sense of life” is manifest in the participants’ everyday experience and behaviour by using themes that consistently resurfaced in the semi-structured interviews as basic clues for orienting the hermeneutical process in question.

Before the interviews, the participants were simply informed that they would be talking about “life in Mecklenburg-Western-Pomerania.” To be sure, a certain “double aspect” (see Przyborski and Wohlrab-Sahr, 2014, 229; Schütze, 1987, 14 f.), according to which the participants can understand their existential orientation “from within” and, in part, also “from without,” is consistent with the guiding methodological assumptions (and was also taken into account by the interpretation of the material). However, the risk of triggering too abstract and ideological statements by the participants rather than getting access to their life experience should be minimised. Thus, the term “sense of life” was avoided in order to prevent the participants from merely confabulating about what they take to be a regionally typical sense of life. The intended phenomenon was addressed in a more indirect manner. Among other things, the interviews touched upon the biographies of the participants’, their lifeworld, social circles, regional elites and prospects.^10^ However, the interviewers adapted the sequence of their questions so that the interview could unfold in close touch with the emerging structure of what seemed to be relevant to the respective participant.

For further analysis and interpretation, the interviewers helped evaluate the quality of the data, e.g., by distinguishing between the participants verbalising generalities or preconceptions on the one hand and the articulation of genuinely felt meaning on the other. The aim was not simply to grasp what the participants wanted to convey but to reconstruct the structures according to which meaning and relevance is organised in each individual case.^11^ Then, structural similarities and homologies between different cases were worked out, critically discussed and revised where necessary.^12^ As part of the multilevel hermeneutical process, the interviewers did not only take the *verbal content* of the participants’ responses into account but also what they were communicating *nonverbally*, for instance, their gestures, affective reactions or how they proceeded when asked to produce a collage focused on the future of their region. At a later stage, the philosophers helped to conceptually refine the explication of the findings. In the course of this multilevel process, our comprehension of the participants’ existential orientations was revised, improved and refined, but is, of course, open to further revision in the light of new evidence.

Although the interviewers avoided the term “sense of life” and formulated their questions as open as possible, a certain priming effect should be taken into account. Given that the participants were informed that they would talk about living in that particular region and that also various questions would suggest a regional context, we may assume that they, at least in part, focused on those aspects of their existential orientation that they thought had communal relevance. That is, the participants were likely prompted to provide appraisals of their situation not only in terms of how it appears to them as individuals but also in terms of how they think it is for “one” living in that region. This effect may have even increased to some extent when the relevant participant sooner or later realised that the interviewers were coming from another region (a major city in Western Germany). Then, by tendency, the participants would register the interviewer as an “outsider” towards whom they attempted to highlight the positive aspects of the region and its inhabitants.

The findings concerning the original intent of this part of the research project have been published in a brochure intended for a broader audience (Großheim et al., 2014). The present article, however, is different in aim and scope as it engages in more theoretical reflections concerning the bodily dimension of the relevant existential orientation insofar as it is manifest in several participants in the interviews.^13^ Thus, I am less concerned with arguments about the way in which these findings could be representative of the people in North-Eastern Germany in general. Rather, what we are supposed to arrive at when applying this methodology in the present context is a morphology of the most common elements of the participants’ individual existential orientations insofar as these orientations relate to the regional context and shape the participants’ normative outlooks.

This claim needs further qualification. The regional “sense of life” or, in more technical terms, the morphology of collective patterns in the participants’ existential orientations, does not refer to a substance above or beyond the relevant individuals. It is rather a common aspect of their background orientation towards the world, in part explicable in terms of having been enculturated in a particular environment.^14^ These commonly discernible patterns are neither homogeneous nor deterministic. We will see, for instance, that the participants may have the relevant patterns in common but also differ with regard to their explicit political views. We will also see that the relevant patterns may also vary among the respective individuals in terms of expressivity, explicitness, bindingness, etc. And, of course, any individual may have other orientations as well. Thus, these patterns are not supposed to totally define the motivational structure of the individuals but to make up a notable part of it.

With regard to the following, a particular limitation concerning the collected data needs to be addressed: Although the affective and embodied dimension of the intended existential orientation was relevant to the design of the investigation right from the start, the importance of factors that are, as I shall argue, best explained in terms of bodily attunement emerged only at a later stage in the hermeneutical process. Presenting the participants with another round of questions more carefully targeted at this issue would have been ideal but was not part of the original design of the investigation. Thus, in the course of the investigation of ethical sensibility as bodily background orientation, I shall flag out some points that have a more hypothetical character, insofar as they seem to be in line with, although not sufficiently clearly supported by what can be established on the basis of the collected data, so that they can only make an appeal to phenomenological plausibility.

## Reconstructing the Quietist Attunement

### Interpreting the Material

In the following subsections, I present a sample of the material in order to reconstruct commonly discernible patterns in the existential orientations of the participants, its bodily dimension, and its role in shaping these participants’ sensitivity to deontic power. I begin by describing two types of participants according to the dynamic of their nonverbal communication (Nonverbal clues to the participants’ orientation toward the world). Then, I present the interpretation of three paradigmatic cases of one type in more detail (Adelheid, Bert, and Christopher) in order to prepare the ground for reconstructing the patterns concerning these participants’ existential orientations (Patterns of a quietist orientation toward the world). I return to the other type of participants at a later stage of the argument (Expanding the pattern beyond the example cases: Contrasts and alternatives).

#### Nonverbal Clues to the Participants’ Orientation Toward the World

An important factor in reconstructing the way in which the participants find themselves in the world is the bodily dynamic in the interaction between them and the interviewer. This dynamic is, among other things, observed in terms of the participants’ postures, gestures, and their demeanour. It provides the interviewer with a preliminary, although partial and inchoate, impression of what they later on methodically work out as a pervasive character of the participants’ general orientation towards the world. There were roughly two types of participants in terms of how the interaction was experienced by the interviewers.

One type (“open”) tends to create an atmosphere of friendly familiarity right from the start, e.g., by warmly receiving the interviewer, providing them with refreshers, such as coffee and cookies, and treating them with trust. Eventually, however, the participant realises that the interviewer comes from the western part of Germany and, briefly, the conversation gets stuck, as if the participant wondered whether they had dared to lay themself open too much. Nevertheless, the friendly and trustful interaction continues so that the brief but palpable halt is moderated by various other aspects of the entire dynamic.

This is different with the other type (“reserved”). The participants of this type do not seem to care about accommodating the interviewer, and by tendency, they merely “react” to the questions without participating actively in the interview. Even experienced interviewers describe them as “not really there” or “a hard nut crack”: “There is something rigid which remains intangible [to the interviewer].” Above and beyond that manifested lack of responsiveness, the conversation gets stuck quite often, although not only for a brief moment. Then, according to the interviewers, “a tomb-like silence” emerges. After the conversation, the interviewer is quickly dismissed. When asked to produce a collage displaying the future of their region as they envision it, this type of participant tends to choose depictions of secluded beaches, panorama views of the typical landscape where almost no people can be seen or pictures with small groups of people as their material. If the collage features people at all, they either appear in terms of harmonious coexistence or, less often, as threatened (e.g., poverty among elderly, protestors thematising the obscure role of the intelligence service in a recent wave of right-wing extremist terrorism) or as threatening (e.g., soldiers equipped for combat). Often, that material was arranged in a rather static manner, and the result often lacked ambition as well as coherence. It seems that the interview, as well as the collage, was taken as a compulsory exercise rather than as a chance for active initiative and playful self-expression. Taken together, the nonverbal data suggest that a predominant character of the way in which these participants engage with and respond to the world is to be described in terms of reserve, withdrawnness, restraint or reticence.

This is a key character of the quietist attunement as discussed in the context of the present paper. The following analysis and interpretation of the empirical material are supposed to further explicate the way in which those who are palpably reserved or withdrawn in their interaction with the interviewer are oriented towards the world more generally. On the basis of this material, we can reconstruct a peculiar pattern of subjective relevance that is common to the more reserved type of participants and that also involves a specific sense of situated normativity. Whether and to what extent this pattern is also a relevant factor in the world orientation of the participants of the first type will be discussed below (Section “Expanding the Pattern Beyond the Example Cases: Contrasts and Alternatives”). In any case, the phenomenon under consideration is more pronounced in the second type of participants (“reserved”); thus, the present analysis focuses on this type. Let us, therefore, have a closer look at three paradigmatic cases of the “reserved” type, Adelheid, Bert and Christopher.^15^

#### Adelheid

Adelheid (26) is about to conclude her studies in geography at the University of Greifswald. For the interview at her home, she would wear a leisure suit (which is why she does not want the interview to be recorded by a camera). She does not provide the interviewer with any drink and also declines the goodies offered to her by the interviewer in their apparently futile attempt to break the ice. From the interviewer’s point of view, the dynamic of the interaction is characterised as “sleepy” because of the perceived lack of responsiveness on Adelheid’s part.

When asked how life is in the region, she states that she feels 100 years behind the rest of Germany, which she also finds nice in some sense. “This place is like the Baltic Sea. It’s always the same water.” – No tide, no change. According to Adelheid, development is something that only takes place somewhere else. Interestingly, though, an obvious candidate for actual improvement in the region, such as the A20, a recently completed motorway being the lifeline of the region, is not conceived as a positive development but rather in terms of the disruption of a prior peace: “We’ve got the A20 now, but it runs right across the natural world.” Here, Adelheid expresses a form of indignation that *prima facie* seems relatively mild and vague. However, the way in which she expresses that indignation is consistent with her overall reservedness (a bitter remark rather than an emotional outburst); the focus of that indignation also provides insight into her pre-reflective orientation in terms of affordance-borne normativity: Rather than perceiving that motorway in terms of valuable infrastructure enhancing the possibilities of moving through the region, Adelheid registers it as a stain on the otherwise unspoiled nature. In the context of her lived environment, the motorway seems to be a foreign object that ought not to be there – in a phenomenologically similar fashion as we may resent a person standing slightly too close to us, or a person breaking the solemn silence we have indulged in, not merely in terms of an unpleasant but also illegitimate disturbance. In Adelheid’s case, in turn, this means that what she perceives as the “natural world” must palpably implicate some sort of normativity pertaining to its preservation: Do not spoil the nature; do not disturb the peace. In the present example, the motorway “running right across the natural world” does not conform to that normativity. Therefore, as concretely responsible persons that otherwise could have filled the role do not occur in Adelheid’s lived environment, that motorway is the object of her resentment or indignation. The pattern of this normative orientation is not restricted to the occasional example of the motorway. It recurs in other contexts as well, for instance, when Adelheid articulates her expectations of political leadership: On the one hand, politicians ought to attract companies in order to help the economic development of the region to catch up with other parts of the country (“100 years behind”), but, on the other hand, the landscape must not be spoiled with industrial areas. The concern for an unspoiled environment is a theme that consistently emerges in many interviews; it is also manifest in many collages, namely in the preferred choice of subjects such as secluded beaches or panorama views on rural landscapes.

In the context of how life is in this region, Adelheid also thematises a commitment to small groups: People from the region – as seen by her – prefer to remain among familiar faces. She states that one is either fully inside or outside. This is supposed to imply that there is no third option between fully belonging to the region and remaining an outsider. This statement also echoes in the interviewer’s feeling of being out of place, the sense of not being accommodated at Adelheid’s place, which is implicit in the (non-) dynamic of their interaction: By tendency, the non-familiar is treated as foreign and kept out. Indeed, “preferring to remain among familiar faces” is, according to many participants, supposed to account for a rather reserved attitude towards foreigners, which is also declared as being typical for the people in this region. A few collages would also express this attitude by framing people either in terms of harmony or in terms of threat (threatened or threatening). Adelheid, however, declares to be positive about the integration of foreigners, but, given her interaction with the interviewer, she seems to understand that the main effort to fit in ought to be on the foreigner’s part. She also states that she still fully belongs to the region but wishes to get out, e.g., by going to Berlin or into the world. This is interesting, insofar as, on the one hand, Adelheid’s plans of moving elsewhere imply that she would not fully subscribe to that preference of remaining among familiar faces and the strong in-group cohesion she ascribes to the typical inhabitants of the region. That is, on a reflective level, she, to some extent, locates herself beyond what she perceives to be the normal. On the other hand, as far as the interaction between Adelheid and the interviewer is concerned, she seems to exemplify what she just described as typical for people from the region. That is, on a less reflective level, she seems more profoundly oriented towards the world in terms of reserve and withdrawnness than she is aware of.

#### Bert

Bert (36) is described as a serious young man. His clothing style, as well as some of his statements, implies a left-wing orientation. At the beginning of the interview, he is very standoffish. Bert is a salesperson by trade, but he quit 2 years ago and is now unemployed and on welfare. He has recently moved away from Berlin, back to the region where he originally came from and where he now lives on his own on the old farm he inherited from his grandparents. The farm is in a bad state, he does not see any job perspective for himself in the region, and he does not have many friends in his everyday environment, for too many of them have moved elsewhere. “I do not have much contact in the village; as far as I’m concerned, the basic attitude there is too right-wing.”

Like Adelheid, Bert believes that people from this region prefer to remain among familiar faces (they are “not easily approachable”). He also seems to have a reflective distance to the attitude he describes, because he relates this statement to his contrastive experiences in Berlin. However, on a less reflective level, he also exhibits traits of a world orientation that is somewhat reserved in character, for instance, in terms of interaction dynamic and his reclusive lifestyle.

Also, like Adelheid, Bert refers to the landscape of the region as something that is unspoiled and should be preserved: “The nature here is more pristine [than elsewhere]; this place is not that obstructed [as compared with other regions].” What Bert intends by “obstruction” is a variation of Adelheid’s perception of the motorway as a foreign object disrupting a prior peace that is connected with the vast rural landscape. The “unobstructedness” of the region is highly appreciated and outweighs the affordances of other places. For instance, Bert clearly sees that the region has fewer job opportunities to offer than places in the western part of Germany have, “but I do not want to live there; everything is so complete there and you have to adjust.” Rather than having to adjust, Bert seems to favour possibilities of freely unfolding himself when he says that he loves this region and the freedom it affords. “In Berlin, you are so constricted; the place here is vast and infinite, and you can do what you want; no one will be bothered.” The vast, unobstructed character of the landscape is perceived as affording the freedom to “do what you want” in a palpable manner as in contrast to other places such as Berlin. The idea is that, in the vastness, one can disappear from the critical gaze of others who might “be bothered,” i.e., react with one or the other mild form of resentment or indignation. The potential of there being such a critical gaze, which is co-intended by this statement, characterises the affordance structure of the regional lifeworld (as Bert perceives it) even further. Those who might “be bothered” are those who just have been described as preferring the familiar; and we have seen that, given Bert’s interaction with the interviewer as well as his reclusive lifestyle, this description is more characteristic of himself than he seems explicitly aware of. In this context, his statement also implies that anything that does not “disappear” in the vastness in the relevant manner will, in the view of those others who might be “bothered,” stick out as unfamiliar and be resented as spoiling the status quo.

#### Christopher

Christopher (22) is a mechatronics fitter by trade but now works as a long-distance lorry driver. In the beginning, he seems shy and polite. In the course of the interview, however, he, with increasing frankness, professes his right-wing views and shows resentment at particular developments in the region. Like Adelheid and Bert, Christopher associates something unspoiled and peaceful with the regional environment in which he finds himself, and he also seems to be sensitive to some felt demand for its preservation. He perceives the region as a chaste entity that is little by little deformed by outside influences, foreign subversive forces. Although he is not able to describe those forces more thoroughly, they conspicuously raise his anger. Christopher adumbrates how the region would look, if only those threatening forces could be minimised, by appealing to imaginations of happy children and flourishing landscapes. Progress and improvement are primarily thematised in terms of renovating already existing buildings and infrastructure, e.g., kindergartens, roads, etc. – the progress of such renovations is actually something which he explicitly takes pride in (“a pattern for other regions, too”). He also appreciates that there are not that many foreign people in his home region and thinks that the right-wing movement is a legitimate reaction to a feeling that he supposes to be prevailing among people from the region, the feeling of having been wronged in the economically and politically turbulent years after the German reunification. Thus, the pattern of perceiving particularly relevant changes in the environment of an individual as problematic deviances from the familiar and resenting them as unwelcome disruptions of a prior peace is also manifest in his case.

Like Bert, Christopher also perceives the vastness of the regional landscape in terms of a felt possibility of freely unfolding himself; yet, he also adds a sense of security and a feeling of belonging to the picture: “The vast sea is beautiful, it conveys the impression of freedom and the quiet rural life gives one the feeling of belonging together.” That impression of freedom is clearly an apolitical one: Rather than struggling and competing with others for the power to act, this is the freedom to be left in peace. Christopher also states that, although he got to know other parts of Germany quite well because of his job, he has never experienced that sense of freedom and warmth elsewhere. In the context of warmth and belonging, he also varies the theme that people from the region prefer remaining among familiar faces and that there is no third option between being recognised as fully belonging to the region and remaining an outsider: It is typical for people from this region, he says, that you first needed to win their hearts, and then they would stand by you, unconditionally.

Secure belonging, being “inside” and being unconditionally recognised among familiar faces allows for a particular form of harshness or acerbity when interacting with one another: “My relatives in [another state in Germany] are polite to everyone, but you never know what to make of this. This is different here. We talk by guess and by gosh [literally: as the gob has grown].” The implication in this context is that “politeness” is regarded as some form of pretence as something artificial and less valuable when compared with things that are supposed to have naturally developed (one’s “gob,” one’s world view, etc.), as something which provokes the speaker’s mistrust and disapprobation (“you never know what to make of it”). Structurally, registering politeness in terms of pretence, and, therefore, devaluing it, corresponds to Bert’s lived aversion to having to adjust himself elsewhere and, probably, even to Adelheid’s seeing no point in dressing a bit more formally for the interview or not providing the interviewer with a drink. It also corresponds to the lack of ambition in the production of some of the collages. In this sense, harshness as a pre-reflectively lived rejection of what one takes to be no more than pretence (“politeness”) is an integral structural element of the world orientation of these participants, and it, in part, accounts for the reserved and withdrawn manner in which they interact with their interviewer. The element of harshness also blends in with other structural elements such as freely unfolding oneself, preferring the familiar and natural purity. Where these latter things are appreciated, “politeness” is in an unfavourable contrast with unfolding in a natural way; it sticks out and is resented as foreign and inappropriate.

### Reconstruction of the Quietist Attunement

#### Patterns of a Quietist Orientation Toward the World

Despite the differences between Adelheid, Bert and Christopher regarding gender, age, education, trade, particular location in the region and the political spectrum, they, by and large, share a specific pattern of subjective relevance, which is also manifest in their way of engaging with the interviewer in particular and their world in general. The most conspicuous tendencies constituting this pattern are these:

1. “tranquillity”: perceiving one’s lived environment in terms of natural purity, tranquillity or peace, and a demand for preservation, while perceiving and resenting particular things that do not fit in with the relevant purity or peace as spoiling.
2. “familiarity”: perceiving those who belong to one’s lived environment as preferring to remain among familiar faces and as being hardly approachable to strangers, while embodying this reduced approachability oneself to a significant extent.
3. “harshness”: perceiving and depreciating particular forms of behaviour that deviate from the context of familiar faces as being a pointless effort, artificial adjustment or pretence, while seeing no problem in a certain acerbity or roughness towards oneself and others (peers as well as strangers are supposed to accept this as a regional norm).

We should not think of these tendencies in terms of isolated units. The material suggests that, for instance, the lived aversion of having to adjust oneself synthesises both a perception of artificiality and pretence (3) and a sense of natural purity and peace (1), i.e., the aspects of pretence and natural purity co-determine one another. Likewise, what or who is perceived as “foreign” in contrast to familiarity (2) is, by tendency, also perceived as “bothering,” i.e., as “spoiling” the peace (1), whereas the peace aspect, in turn, is registered in terms of a *prior* state and thus is inextricably connected with the relevant sense of familiarity. In a similar manner, the readiness to reject pretence (3) co-determines the preferred familiar environment (2) but cannot be properly understood without the sensed security of belonging to that environment. Thus, rather than, for instance, conceiving of those tendencies in terms of contingently combinable attitudes, we should think of them as interdependent aspects of a consistent and coherent *holistic style* of engaging with the world.

This overarching style is, among other things, manifest in temporary attitudes, for instance, towards the interviewer or towards tasks or particular topics of the conversation. Ratcliffe (2020, 257) points out that styles in this sense may provide contexts from which more localised experiences such as episodic emotions arise. This seems to be the case when the participants, according to a specific pattern, express their resentment to particular developments. What is more, in contrast to completely unconscious dispositions to perceive and act in a particular manner, their style of engaging the world seems to involve a peculiar phenomenology, *a sense of what it is like* to be oriented in this way, albeit, by and large, a pre-reflective one. This experiential dimension is referred to when, for instance, one reports that one *feels* 100 years behind and somehow *likes* it this way or that one *loves* the freedom *palpably afforded* by one’s lived environment, or when one links the perceived character of the surrounding landscapes with *impressions* of freedom or *feelings* of belonging. Furthermore, the relevant experiential dimension is there, when the speaker contrasts the impression of these landscapes or the perceived quirks of the people from that region with their *experiences* of other places, or when they refer to a *feeling* of having been wronged. Again, these and other references made by the participants are best explained as integral aspects of a pattern of subjective relevance that they, by and large, have in common. Thus, it seems warranted to interpret this pattern in terms of a specific *experiential* style.

As indicated before, an inquiry more carefully targeted at the bodily dimension would have been desirable in order to further enrich and validate the present interpretation. However, we have seen that the vast, “unspoiled,” rural landscape, consistently mentioned in terms of an atmospherically palpable character pervading the participants’ lived environment, is often thematised as a particular affordance structure: The perceived quality of that environment seems to afford certain ways of doing things and not others. Moreover, the material suggests that the landscape, at least in the perspective of the participants, “scaffolds” their affectively coloured style of engaging the world (see Colombetti, 2017, 1,445 f.). The lived, as well as explicitly reflected, preference of the participants to return to or stay in the region functions in the fashion of selecting, inhabiting and maintaining a long-term “affective niche” that environmentally embeds their common subjective-relevance pattern (ibid., 1,444). These findings, as well as the consistent and coherent style in which the participants engage and experience the world – a style manifest in their gestures and demeanour, their interaction with the interviewer – suggest that the reserved, quietist world orientation is *embodied bottom-up* and give high phenomenological plausibility to interpreting it as some form of *bodily* background orientation.

Taking into account the holistic, embodied and experiential character of the orientation towards the world explicated so far and its implications regarding the affordance structure, the congruities with the category of *existential feeling* are striking. In line with this category, I suggest conceiving of this orientation as the participants’ *bodily attunement*, an attunement they are, by and large, pre-reflectively aware of. The attunement may be labelled as “quietist” in that it is related to the pervasive atmospheric quality of their lived environment (tranquil, familiar, rough). It can be approximated by the outlined tendencies of feeling comfortable with tranquillity (1), being hardly approachable to strangers (2) and avoiding adjustment to “artificial” norms (3).

Like existential feelings in general involve openness to certain kinds of possibility, the quietist attunement entails responsivity to a certain type of *ethically significant affordances*. The particular sense of demand may vary from individual to individual or from occasion to occasion. For instance, due to his job as a long-distance lorry driver, Christopher might not be expected to condemn the new motorway,^16^ which is the particular target of Adelheid’s resentment, nor is Bert likely to agree with Christopher’s open resentment towards foreigners as such, etc. Likewise, one and the same individual may, on one occasion, resent the obstruction of the landscape and, in a different context, be bothered by whatever or whomever sticking out as spoiling the prior peace. However, the subjectively felt demands that make all these instances of resentment intelligible are of the same type in that they have a specific structure in common: They present a holistic synthesis of preserving tranquillity, sticking with the familiar and rejecting pretence as the appropriate response to a pervasive atmospheric quality of the participants’ lived environment. In the context of individually variable circumstances, that common structure imbues the lived environment with a specific *affordance-borne* or *situated normativity*, against the backdrop of which particular objects, persons or developments manifest themselves as inappropriate, perhaps even as offending (or pretentious) disruptions of a prior natural purity and peace. Likewise, action programmes directed at integrating the relevant disruptive element into the familiar framework, allowing it to “disappear” in the vastness or rolling back a supposedly spoiling development, appear, at least by tendency, as legitimate or even called for. Please note that the claim is not that this situated normativity totally determined the motivational structure of those three participants, but that it palpably shapes that structure in all three cases. It informs a relevant part of these participants’ (inter-)actions insofar as they inhabited a different space of possibilities and, thus, were likely to act in a different manner, if they were not responsive to this normativity.

In this sense, the quietist attunement involves an ethical dimension, a sense of how things ought to be and of how to conduct appropriately. In other words, the attunement organises an individual’s recognition of desire-independent reasons for actions that, in a given context, seem suitable for avoiding pretence, for respecting the tranquillity, etc. The thusly attuned individual is responsive to the particular situated normativity not only in terms of content (i.e., the salience of *what* ought to be done or avoided) but also in terms of motivation or at least recognition (i.e., the salience of *that* one ought to do or avoid doing it in terms of feeling committed to a particular way of conduct, feeling entitled to rebuke others accordingly, etc.). Being this responsiveness, the attunement is a specific aspect of the individual’s overall disposition to recognise deontic power (in terms of *what* and *that*) – an aspect that is inextricably connected with, although not completely reducible to, other aspects defining the relevant disposition such as more abstract doxastic states, narratives, etc.

#### Expanding the Pattern Beyond the Example Cases: Contrasts and Alternatives

In the three cases considered, the tendencies (1)–(3) define a peculiar, ethically relevant attunement to a pervasive quality of the relevant participants’ lived environment. The remainder of the material collected suggests that these tendencies are also part of the pattern of subjective relevance in many other participants regarding both their interaction with the interviewers and their verbal responses. However, the relevant tendencies are clearly less pronounced but rather moderated by there being other noticeable traits as well, while such moderation seems less distinct in the interactions with Adelheid, Bert and Christopher. For instance, the brief but palpable halt of the conversation observed in the interaction with the more open and accommodating type of participants can be interpreted as a moderate echo of that reserve towards strangers, which is a tangible character of the world orientation described in the previous section. It is noteworthy that this halt regularly occurs at precisely that point of the conversation where the participants seem to identify the interviewer as a member of an existentially relevant out-group, the relation to which is conflict-laden. Thus, lacking familiarity emerges as a problem, if only briefly and in a very particular context. After that brief moment, the participants of the “open” type seem capable of activating various other resources so that the interaction continues in a friendly atmosphere.

The pattern recurs more explicitly in the verbal responses. Like in the cases of Adelheid, Bert and Christopher, highlighting natural vastness, purity and peace as the regionally peculiar quality of one’s lived environment is quite common among the participants.^17^ Some participants also express a rather general preference for the familiar.^18^ Others contrast the reserved attitude with rather resentful characterisations of those who apparently do not comply with that attitude and who, therefore, are perceived as less committed to small, familiar groups and the situated normativity of reserve.^19^ Such resentful characterisations (“obstreperous,” “fancy-schmancy,” “acquaintances at best”) suggest a tendency similar to that of perceiving and rejecting “politeness” as mere pretence. Some would even bring in a slightly different perspective on the matter and state that they had to work hard to overcome their tendency of avoiding what others might take as pretence in order to succeed in their relevant professional context.^20^ Yet others, when finding themselves praised by the interviewer for their activism, tend to immediately deflect the praise by stating that their accomplishments are nothing to speak of and thus acknowledge, at least indirectly, the normativity of reserve by seeking to avoid “pretence.”^21^

This brief survey of the material collected suggests that the tendencies (1)–(3) are not limited to the paradigmatic cases of Adelheid, Bert and Christopher. The verbal responses of many other participants also point out the natural purity of the landscape and the peaceful environment, show a commitment to smaller groups, as well as to keeping things calm and quiet, and also tend to reject pretence when characterising life in this region. However, the relevant tendencies are also moderated by other aspects – e.g., more proactive traits – so that they constitute less specific a character of how these participants are oriented towards the world as compared with the cases of the “reserved” type.

In some participants, there is clear evidence that the tendencies (1)–(3) are as holistically synthesised as in the cases of Adelheid, Bert and Christopher. For instance, a participant’s characterisation of the region as “sleepy” is immediately followed by the claim of there being “no obstreperous folks” so that a connection between the tranquillity of the landscape and the situated normativity of reserve is presented as a matter of course. We also find evidence that many participants contrast their descriptions of how life is in the region with their experiences with other places, express that they find coping with issues outside the familiar context challenging or express some mild forms of resentment when referring to those who do not seem committed to the quietist normativity. In these cases, an experiential dimension is involved in the way in which they articulate living in the region along the lines of the tendencies (1)–(3). And if the given interpretation of the brief halt in the otherwise friendly interaction is correct, then there is also a bodily dimension involved in the way in which these participants are attuned to the situated normativity of familiarity and reserve, although they certainly seem less thoroughly attuned in this way than, for instance, Adelheid, Bert and Christopher. If this is correct, then we may also regard the tendencies (1)–(3) as characteristics of these participants’ bodily attunement to a pervasive quality of their lived environment. However, as compared with the cases of Adelheid, Bert and Christopher, we should also think of these attunement-defining tendencies as being more thoroughly moderated with a variety of other aspects that outbalance them rather than overriding them completely. In other words, the quietist attunement characterises the existential orientations of these participants to a smaller but, nevertheless, significant extent.

The relevant attunement-defining tendencies seem to make up a commonly discernible, ethically relevant aspect of the existential orientation of various participants. This may have interesting socio-ontological implications, for instance, regarding the conception of collective attunements, which cannot be pursued here. In the context of this paper, the more relevant point is that the pattern so clearly pronounced in the cases of Adelheid, Bert and Christopher reproduces in a modified but yet regular way in other cases, too, which enriches our understanding of the phenomenon. The attunement in question can be more or less fundamental, it can be embodied in a relatively pure manner or enriched with other elements, an individual can cultivate it or emancipate from it, etc.

## Discussion

The quietist attunement is pronounced to various extents among several participants. *To what extent exactly* the existential orientation of a particular participant is constituted by this attunement cannot be established on the basis of the collected material. However, it is highly unlikely that the existential orientation of any participant is constituted by the relevant attunement alone. What can be established, though, is *that* the attunement seems to play a sufficiently relevant role in the orientations of many participants and *how* these participants’ normative perspectives are organised, *insofar as* they are attuned in the relevant manner. If, in contrast to the cases presented in the previous section, the pattern of a quietist attunement is hardly discernible in an individual, then the relevant attunement cannot be ascribed in any meaningful way to them.

If the preceding interpretations and reconstructions are valid, the quietist attunement involves an ethical dimension. In the cases discussed, both the salience of *what* one ought (not) to do and the sense *that* one ought (not) to do it are notably shaped by the attunement to a pervasive quality of the relevant participants’ lived environment, a quality implying the traits of tranquillity, familiarity and harshness. The attunement in question is embodied in the comportment of the participants when interacting with the interviewer, and, to some extent, in their lifestyle and in particular ways of engaging in a task. It is also very likely that the attunement is connected to a particular phenomenology of bodily feeling, for instance, in resenting what the participants take to be a violation of the oughtness implied in their sense of tranquillity and familiarity. In this sense, the quietist attunement can be conceived of as an ethically predisposing, common (perhaps even shared) aspect of these participants’ bodily background orientation. As we have seen, this aspect may be more pronounced in some participants than in others. It may also be more pronounced in some situations and less so in others. What is more, we should not expect this aspect to be deterministic in the sense that it irresistibly controls the actions of any individual. However, as the collected material suggests, the sense of commitment to the situated normativity of reserve is not easily passed over by those attuned to it.

That the quietist sense of normativity seems to be connected to comparatively mild forms of resentment where a norm is perceived as not having been met (yet) by relevant others might imply that the respective norm is not felt as unconditionally binding as, for instance, moral core norms such as “Thou shalt not kill.” A violation of the latter in one’s lived environment would certainly provoke stronger forms of resentment than those observed in the context of the present investigation. However, when compared with other forms of affectivity – e.g., the “directed (dis)content” that underpins the situated normativity guiding the work of Wittgensteinian craftsmen (Rietveld, 2008, 980 ff.; see Wittgenstein, 1978) – even mild forms of resentment-like feelings seem to imply a sense of normativity that is different from the sensitivity to instrumental or aesthetic norms. And, although the quietist normativity does not seem to be as unconditionally binding as moral core norms, it is binding in an ethically relevant manner as it seems to relate to the status of a person, to being worthy of the respect of other members of a community that, at least from the perspective of the relevant participants, matters. Moreover, it stands to reason that there are other flavours and layers of bodily background orientations that are connected with more full-blown forms of ethically relevant feelings, such as resentment, shame, guilt or indignation and which, in principle, could be explored with similar methods, although with a different focus of inquiry.

Throughout the investigation, I have explicated the peculiar sensitivity to ethically significant norms by employing the language of deontology (deontic power, commitment, “oughtness,” etc.). By this, I do not mean to adopt a specific approach to ethics in general or to exclude other approaches, such as, for instance, those based on virtues (e.g., McDowell, 1998). Rather, I intend to tie in with socio-ontological discussions of deontic power (see Searle, 2010). I also prefer this terminology for reasons of descriptive aptness as it makes it easy to take into account the tension between what one desires and what one feels that one ought to do.^22^ Thus, if it is plausible that the quietist attunement investigated here implicates a sensitivity to particular forms of deontic power, we may categorise it as a deontological orientation or, given the close relation to Ratcliffe’s concept of existential feelings, as a *deontological feeling*.

The analysis has broader theoretical implications. It exemplarily highlights a relevant aspect of our normative background and recommends the category of deontological feeling as a powerful tool for research in moral and social psychology. If the given account of the quietist attunement is, by and large, correct, it can help elucidate how deontological feelings are supposed to function in general. It indicates what needs to be in place whenever we recognise something as our obligation or our rightful claim on others: A subtle sense not only of how things *are* in the world but also of how they *ought* to be. Being bodily responsive to a particularly ethical type of solicitations, deontological feeling makes up a relevant part of the normative background of a person or a collective. However, as compared with, for instance, affectively laden “moral foundations,” such as “loyalty,” “authority,” “purity,” etc. (see Haidt, 2012), deontological feelings seem more embedded in holistic forms of life and, as the cases of Adelheit, Bert and Christopher imply, less correlated with explicit statements concerning the individual’s endorsement of particular policies.

Since deontological feelings, by definition, are part of the affective background of one or more persons, they are less easy to pin down than, say, momentary surges of anger or shame. Therefore, experiences of difference are the most common way in which we can become explicitly aware of deontological feelings, occurring, for instance, when we find something problematic about them or when our normative sense is critically challenged or is challenging another normative background.^23^ Extant accounts concerning the normative dimension of existential feelings of guilt, anger and shame in psychopathology (see Ratcliffe, 2010; Bortolan, 2017) already point in this direction.

## Conclusion

I have suggested that our normative background orientations, against the backdrop of which something emerges as a compelling reason or an obligation, have an irreducible bodily-affective dimension. Adapting Ratcliffe’s notion of existential feelings and the way in which such feelings are correlated with our bodily sensitivity to practical possibilities, we may speak of *deontological feelings* as bodily background orientations towards the world in terms of which we are sensitive to various forms of deontic power.

By referring to qualitative empirical findings, I have investigated the nature of a particular deontological feeling that we may term as “quietism.” Quietism is a bodily attunement to a pervasive quality of the lived environment of the relevant individuals. It notably shapes both the salience of *what* one ought (not) to do as well as the sense *that* one ought (not) to do it. The analysis has broader theoretical implications in exemplarily highlighting a relevant aspect of our normative background that is underexposed in current discussions of both social theory and embodied cognition.

## Data Availability Statement

The raw data supporting the conclusions of this article will be made available by the authors, without undue reservation.

## Ethics Statement

Ethical review and approval was not required for the study on human participants in accordance with the local legislation and institutional requirements. The patients/participants provided their written informed consent to participate in this study.

## Author Contributions

The author confirms being the sole contributor of this work and has approved it for publication.

### Conflict of Interest

The author declares that the research was conducted in the absence of any commercial or financial relationships that could be construed as a potential conflict of interest.

## Footnotes

^1^In the context of this investigation, I work with a broad notion of the ethical that not only comprises moral core norms (e.g., thou shalt not kill), but also encompasses social normativity.

^2^Please note that this label is not intended to suggest any historical parallels to religious movements going under the same name nor to subscribe to a particular meta-ethical position concerning the existence and ontological import of moral facts.

^3^This is not supposed to mean that one would constantly have to feel the demand of keeping one’s promise in order to recognize one’s obligation to do so as ethically binding. Rather, if one consistently fails to feel the demand whenever that promise is salient in one way or another, one does not understand the obligation in any ethically meaningful way. In this case, to use the terminology of a different, though in some respects similar approach (Michael et al., 2016), one cannot be said to “honor the commitment” implied in the promise, because one is not appropriately motivated to keep it.

^4^A more detailed account of the meta-ethical significance of the perspective of an agent must wait for another occasion. However, see Helm (2017) for a sophisticated account of how to ground the warrant of ethical judgment in the structure of shared subjective meaning (“projectible, rational patterns” of caring manifest in one’s emotions, desires, judgments, actions, etc., see ibid., 36, 39).

^5^See Dewey (1931, 99). Dewey argues that any existential proposition such as, for instance, “the native American was stoical” or “the stone is shaly” ultimately refers to a particular pervasive quality against the backdrop of which “native American,” “stone,” “stoical” or “shaly” can make sense (94 f., 97 f.). A pervasive quality constitutes the scope and context – the “situation” – by the implicit cues of which any thought, insofar as it is methodic and distinct from the lose association of ideas, is oriented (96 f.).

^6^The agent’s responsiveness to ethical normativity is grounded in their personal experience as well as in their personally appropriated experiences handed down by culture. By and large, the latter thematise intolerable deviances from collectively coping with historically variant conditions (Haidt, 2012; Tomasello, 2016). The “moral” of those experiences can be explicated, codified, abstracted from particular experience, generalised and then be used for metaphysically grounding deontic power (e.g., in a concept such as property right) (see Schmitz, 1973, 23 f., 67 ff., and 81 ff.).

^7^Affective anticipation differs from abstract and non-affective expectations (“I probably would feel *E*, if I *φ*-ed") in that it is part of the sense of what the situation palpably affords. In a manner similar to feeling the dangerousness of a dog “through” a sense of one’s body as “rigid and ready to be attacked” (see Colombetti, 2014, 122, 126), one feels the bindingness of a demand “through” that vague sense of constraint as an implicitly felt preparedness for shame-like feeling (see Nörenberg, 2020, 204). Within the present theoretical framework, completely non-affective expectations could not play the role of explaining how we actually understand a norm as ethically binding. Interestingly, some of the examples quoted by Michael and colleagues in their analysis of the sense of commitment could be interpreted in terms of affective anticipation, e. g., the reactions of people being called and asked for money by a confederate (Michael et al., 2016, 7; see Gaertner, 1973).

^8^See Charlesworth (2009) for a related approach which, although its central category is certainly that of class, *en passant* also explicates a regional sense of life (see 150 ff.).

^9^See Appendix A for the sampling of the participants.

^10^See Appendix B for a more detailed list of topics and questions.

^11^Whether the participants’ responses are objectively correct, is not relevant to this method. For instance, statements about the unspoiled nature as the central character of the region may be contested from an environmental engineer’s point of view. However, such statements, especially when they are consistent with other hermeneutical clues, help us to reconstruct the pattern of what seems to be relevant to the participant.

^12^This method in qualitative research reflects what classic phenomenologists mean by “eidetic variation” and its intersubjective validation (see Gallagher and Zahavi, 2012, 29 f.; Kvale and Brinkmann, 2009, 26 f., 241–266).

^13^In part, this article refers to the material presented in Großheim et al. (2014, pp. 32–46), but, given its different aim and scope, to a very large extent, the material presented in this article is previously unpublished.

^14^To what extent this aspect is not only a *common* feature among the relevant individuals but also *shared* by them in a stricter socio-ontological sense is an interesting question which I cannot pursue here. For an account of how bodily background orientations can be shared, see, e.g., Weber-Guskar (2017) and Nörenberg (2018).

^15^The names are fictional to preserve the anonymity of the participants.

^16^Additionally, in the region where Christopher lives, the relevant section of the motorway had already been in use long before the section in Adelheid’s region was completed, therefore it may not be as recent a change in his lifeworld as it is in hers.

^17^“Here, things aren’t that regulated yet and still are underexplored. There are still widely untouched paths in the woods.”; “Here in Mecklenburg, one has one’s peace. Things will stay as they always were. […] What is keeping me in Mecklenburg-Western-Pomerania is my family, my house, and the immutable landscape.”

^18^“It’s a constant coming and going; I’d be happy with a stable place for me.”; “Today, everything has become so complicated; you have to take care of so many things, whether it is education or supplementary child allowance. Back then, there was one claim form and everything worked.”

^19^“In Mecklenburg-Western-Pomerania, everything is somewhat sleepy – even the turnaround was. No one realized that anybody would be locked up [by the socialist regime]. There were no obstreperous folks in Mecklenburg-Western-Pomerania. That is not our mentality. […] We here in Mecklenburg are no fancy-schmancy society. We are honest and down-to-earth – not as upstage as they are in the West.”; “I once asked my friends in Frankfurt [a city in western Germany] whether they would help me with moving. Suddenly, everyone was otherwise engaged; none of them helped me. I then realized that they were no friends, they were acquaintances at best… Such things could never happen here.”

^20^“For the first time I had to engage in sale promotion and bell ringing. That’s not my nature. Politeness was not part of our education.”

^21^“There are so many people who get something up […].”

^22^Such a tension is, for instance, manifest in the testimony about having had to overcome one’s tendency of reserve in order to succeed with one’s enterprise (“bell ringing […] not my nature”; See also Nörenberg, 2020, 193).

^23^To some extent, the inquiry into the regional “sense of life” draws on such experiences of difference, since the interviewers came from a comparatively urbanized region in the western part of Germany and reported one or the other mild form of cultural shock on their part regarding the interaction with some participants.
