# Supplementary material for: Deontological Feeling: The Tranquil, the Familiar and the Body
Source: Front Psychol. 2021 Jun 29;12:662675. doi: 10.3389/fpsyg.2021.662675 (PMC8275923; doi:10.3389/fpsyg.2021.662675)
Supplement: Supplementary file 1 [file Data_Sheet_1.docx]

Deontological Feeling: The Tranquil, the Familiar and the Body

**Appendix A: Sample**

Number of participants: 26

Locations: 11 participants come from cities (Rostock, Greifswald)

15 participants come from a rural environment

Gender: 50% female, 50% male

Age: 20–75

Age groups:

9 participants between 20 and 29 years

8 participants between 30 and 49 years

9 participants between 50 and 75 years

Education/profession graduation from school and higher education: diverse

up to 5 unemployed

various occupational categories (at least 1 entrepreneur, 2 teachers, various employees)

Place of birth 21 participants born in Mecklenburg-Western-Pomerania

(including returnees)

5 newcomers (moved in between 1990 and 2000)

Family status 50% with children (including empty nesters)

50% without children

Home interviews 20 participants were interviewed at their homes

**Appendix B: Questions**

The interviews were prepared and conducted by two staff members of the Cologne based *rheingold*-institute trained in psychology and depth analysis.

1. Warm Up

The relevant socio-demographic data are gathered, an atmosphere of mutual trust is to be generated, the participant is attuned to the subject.

- Gender and age
- Since when were you in Mecklenburg-Western-Pomerania? In which regions?
- Education and professional advancement
- Family status/number of children
- Living and housing situation
- Hobbies, memberships (associations, parties, etc.)
- Income

The interviewer explains that they are not interested in blanket judgment or political statements, but in personal experiences that the participant has made in Mecklenburg-Western-Pomerania. The participants are ensured that their statements are anonymised.

2. First associations, subsumptions, matters of course (including clichés, prejudices – what do others think of us?)

The first steps into the subject are to be made by way of first associations. The focus is on typical and unique traits – both in one’s own perspective as well as in the perspective of others.

- What does spontaneously come to your mind when you think of Mecklenburg-Western-Pomerania?
- What is typical and distinctive of this federal state?
- What is the difference between Mecklenburg-Western-Pomerania and other federal states in East and West Germany?
- What places are typical of Mecklenburg-Western-Pomerania, what places are less typical?
- How would you characterise life in Mecklenburg-Western-Pomerania? What is distinct about it? By which realities is it influenced?
- How is life in Mecklenburg-Western-Pomerania?
- What is distinct about a typical resident of Mecklenburg-Western-Pomerania?
- What is a particular capability or skill of the people Mecklenburg-Western-Pomerania? What can people of Mecklenburg-Western-Pomerania be proud of?
- What are their weaknesses? What are you less proud of or even ashamed of?
- Who is a typical celebrity from Mecklenburg-Western-Pomerania (contemporaneous or historical person)?
- What do people from other federal states in East and West Germany think about Mecklenburg-Western-Pomerania and the people here? What prejudices are frequently heard?
- Which ways of behaving are typical of people from Mecklenburg-Western-Pomerania? To what extent do you behave like this yourself?
- What does this reveal about the federal state and its residents?
- Which of these features do you like, which of these do make you happy – and which do not?
- What does someone who moves to Mecklenburg-Western-Pomerania expect from their new homeland? What does they actually experience in the first months here?
- What would Mecklenburg-Western-Pomerania look like if it was a planet of its own?
- Have there been events in the last ten years that changed Mecklenburg-Western-Pomerania? What would you link this to?
- How would Mecklenburg-Western-Pomerania have to change in order that other federal states raised their hat to Mecklenburg-Western-Pomerania?
- What peculiarities must never be lost by the people here?

3. Tradition and history (Where do I come from? Self-concept)

Questions concerning the participant’s life, homeworld, home and belonging are discussed. The personal relationship to Mecklenburg-Western-Pomerania is in the focus.

- What about your childhood? Where did you grow up? How did you feel there?
- What typical situations or experiences with the region and the people there do you remember from childhood and youth days?
- How often did you move to other regions? Where?
- In which life phases or regions did you have a sense of belonging?
- In which regions or cities outside of Mecklenburg-Western-Pomerania are you staying on a regular basis? Where (else) do you feel you belong? What is the difference between these regions and Mecklenburg-Western-Pomerania?
- Which places or regions (place of birth, hometown, homeland, residence of friends, partners, etc.) are just as important as or even more important than your current residence?
- Why are these other places (more) important?
- What are key moments in your life regarding any region?
- What personal experiences or events have left their marks on you in the last years?
- How do you experience and evaluate the “German unity” today? What is your position?
- In which contexts do distinctions between East and West Germany play a role in your life?
- How long will it take until the unity is truly achieved?
- What does come to your mind regarding your homeworld? Where is home?
- What associations come to mind, which images and emotions are linked with that concept?
- In which situations did you experience a sense of belonging in Mecklenburg-Western-Pomerania? What exactly was this like? When, where, with whom? How did it feel? (acurate description of the situation, course of experience, one’s own actions, involved parties, reverberations, etc.) What is appealing about this feeling?
- What does your home mean to you? Which images and emotions are linked with it?
- When do you experience something like a home in Mecklenburg-Western-Pomerania? What is constitutive for this home?
- When or where did you not feel at home in your life? What did you miss then? What would you link this feeling to?
- If you had to explain your personal relationship to Mecklenburg-Western-Pomerania to someone else, what would you say?
- How does your behaviour change, when you are traveling outside of Mecklenburg-Western-Pomerania?
- How does this behaviour differ from your behaviour when you are back in Mecklenburg-Western-Pomerania? Is there something you could not do or experience while you were travelling outside of Mecklenburg-Western-Pomerania that you can now?
- What do your parents think of Mecklenburg-Western-Pomerania? What opinions do (or did) they hold regarding home or Mecklenburg-Western-Pomerania? What experiences did they make?
- On which occasions do you talk with your parents, your partner, your children, etc., about Mecklenburg-Western-Pomerania? Which issues are discussed? When was the last time… (description of the situation)?
- Which hopes and fears do you have when thinking about the further development of Mecklenburg-Western-Pomerania?
- Which experiences or images possibly do prevent you from feeling comfortable in Mecklenburg-Western-Pomerania?
- Would you like to acquire citizenship of another country? Which country? Why?

4. Creative power/leadership (who or what has the power to lead?)

The sense of life depends on both the particular individual and a supra-individual societal situation. The single individual can delegate responsibility to others. Therefore, it is central to inquire after who is bearing the responsibility for Mecklenburg-Western-Pomerania and which developments are regarded as a pattern for other regions.

- Which influences (powers, forces) do you think are determining the developments in Mecklenburg-Western-Pomerania?
- How would you describe the role played by parties and politicians? In what way are politicians responsible for the developments? What do you expect from politics or politicians?
- Who is actually bearing responsibility in Mecklenburg-Western-Pomerania? Who should bear it?
- Who else, which groups or which societal powers are decisive for the further development of Mecklenburg-Western-Pomerania?
- Where do these powers come from? Do they know Mecklenburg-Western-Pomerania or did they come from somewhere else? What interests do they have?
- Who does stand up for Mecklenburg-Western-Pomerania? Who is interested in developing this state in a fruitful manner?
- How do you see your own role in this context?
- Who is responsible for successful or unsuccessful developments in Mecklenburg-Western-Pomerania?
- Who or what is preventing that “reasonable” developments are pushed through or implemented in Mecklenburg-Western-Pomerania?
- Where are the old elites? Who has left Mecklenburg-Western-Pomerania, who is still there?
- How many people in the current elites (politics, economy, culture, universities,…) have come from the western part of Germany? What is that like to you?
- Do you see sufficient efforts for a successful future of this state? In which areas are there successful developments?
- In which areas do you perceive to be a lack of impulses and activity? Do you perceive a slowing down in the development of Mecklenburg-Western-Pomerania? What are the reasons, who is responsible?
- What kind of activity could help advance Mecklenburg-Western-Pomerania?
- What could you yourself do? Have you been active somewhere (associations, demos, reader’s letters…)? What was this like? Did you have any success? What are your plans in this regard?
- With regard to which points are you ready to bring sacrifices for a new development? With regard to which points are you less ready?
- Example unemployment: What is the reason for the high numbers of unemployment? Do you see a (positive) development in the labour market? How would you back up your assessment?
- How is it that efforts for change and development do not have the intended effect? Is there something that is blocking the developments?
- Which powers actually do advance something in the state? Where is progress, where stagnation?
- Where and how can powers for new developments and new drive be mobilised?

5. Future prospects (What is the overall tendency of the current developments?)

- What will Mecklenburg-Western-Pomerania’s further development look like? What will Mecklenburg-Western-Pomerania look like in five years from now? What are your hopes? What are your fears?
- What will Mecklenburg-Western-Pomerania look like, if the current developments simply continue? Which images come to your mind, when you think of Mecklenburg-Western-Pomerania’s future ? What does this feel like?
- Where can you see such development already now? Are there examples that could make Mecklenburg-Western-Pomerania’s future intuitively accessible?
- What will communal life look like ten or twenty years from now? What will other aspects addressed so far (e.g. unemployment, …) look like?
- If you think of Mecklenburg-Western-Pomerania as a planet – what will this planet look like in ten years from now?
- What are your dreams (including wishful dreams) regarding Mecklenburg-Western-Pomerania? What is your wish for this region?

In this step of the exploration, half of the participants are also asked to unfold their future prospects regarding the region by creating a collage. The participants are provided with a selection of various magazines which they can use as a resource in order to freely choose and arrange pictures and photographs for a collage that should convey their vision of Mecklenburg-Western-Pomerania’s future.

The interviewer asks the participant to comment what they is doing. After having finished the collage, the participant is asked to comment on their selection and arrangements of the various parts.

- What are you particularly interested in (hobbies, interests, projects, etc.)? In which areas do you prefer to be active in everyday life?
- What issues do you keep track of when watching television, surfing in the internet, reading magazines or books?
- What are frequent talking points among your colleagues or friends? In which way are these points important to you?
- With regard to which point would you like to be more (pro-)active?
- What are your wishes and expectations regarding your own future? What aims and ideals do you have regarding your life?
- What did you “achieve” in life so far, what did turn out well?
- What else do you want to achieve?
- How are family life and professional life ideally supposed to go on?
- What are your guiding principles in life? What role models do you have? What is guiding you?
- What do you want to achieve within the next five years? At which points do you want to make progress in your life? Which role is played by your profession, your activities in associations or with your friends?
- In which areas are you planning for changes (partnership, desire to have children, career, hobbies, etc.)?

6. (Conceivable) steps in the development (What am I up to? Desire to have children, career, etc.)

- What piece of advice for life would you give your children with regard to Mecklenburg-Western-Pomerania’s future prospects?
- Which political ideas concerning Mecklenburg-Western-Pomerania did inspire you in the past? Which ideas concerning the economy? What exactly did inspire you?
- What else does inspire you (politics, economy, science, sports, other people, etc.)?
- Do you still have dreams concerning your life? What do they look like? Can you go for them in Mecklenburg-Western-Pomerania? Would going for your dreams be easier elsewhere?
- Are you planning to relocate elsewhere or working in another job?
- What is it that keeps you in Mecklenburg-Western-Pomerania? What are the advantages of living here compared to living elsewhere?

7. (Work) environment, skills and strengths (What abilities do I have? What are my skills?)

- What challenges do you see in the near future for yourself? What do you need to do in order to master them?
- Do you feel prepared to go along with the developments in Mecklenburg-Western-Pomerania? At which points do you have fears? How could these fears be calmed?
- Which developments in Mecklenburg-Western-Pomerania would you yourself support with activism? What ideas in politics and economy do inspire you to such an extent that you would get involved yourself?
- What contribution are you ready to make? What would make it easier for you to make that contribution?
- What is discouraging you from contributing?
- Do you see the possibility to achieve something for Mecklenburg-Western-Pomerania by changing your own life? Or is merely reacting to influences from outside the only thing to do?
- What needs to happen with society in order that Mecklenburg-Western-Pomerania can get into a new development?
- What (material or non-material) tools are needed by Mecklenburg-Western-Pomerania in order to engage with new developments? Is it more money from outside or more stimulus from inside, or what else?
- What is your ideal concept to deal with unemployment?
- How can unemployment be overcome? What strategy do you regard as efficient?
- At which points did you make changes in your life so far? Can you give examples?
- How did these changes come about? What was the initial situation? What was the concrete inducement to change something?
- What did the immediate consequences look like? What was this like for you?
- Would you make the same decisions again, would you make through the same developmental process again, or would you prefer to have something changed?
- Do you have a strategy in order to deal with changes? How would you describe that strategy? Are some of your strategies also tried and tested?

The participants are provided with print material (marketing campaign for the region, campaigns of politicians and parties in the region, etc.).

- How does the participant react?
- What does this example tell about Mecklenburg-Western-Pomerania? What do you make of this? What is fitting for Mecklenburg-Western-Pomerania, what is not fitting at all?
- What (right or wrong) impression does this convey to persons from outside of Mecklenburg-Western-Pomerania?
- Which images, themes, or statements would fit better? What message about Mecklenburg-Western-Pomerania would be more appropriate?

8. Conclusion and outlook

The last step of the exploration is supposed to give the participant the possibility of wrapping up the interview.

- Which aspects of the interview have been particularly interesting? What was particularly thought-provoking?
- What are the central points that have been thematised during the interview?
- Does the interview have an effect on your previous perspective on Mecklenburg-Western-Pomerania? If so, which one?
- What is your estimation of the future of Mecklenburg-Western-Pomerania and the people living here? What developments do you wish for yourself?
- At which points or in which situations do you have fears concerning the development of Mecklenburg-Western-Pomerania?
- In which areas do you expect pleasant, sustainable improvements?
- In which areas would or could you become (more) active in the future? Or which person or group should, by proxy, become more active?
